# Transition From a High‐Sugar and Butter to a Standard Diet Leads to Cecal Dysbiosis, Disrupts Intestinal Homeostasis, and Favors Increased Ethanol Consumption and Preference

**DOI:** 10.1096/fj.202502123R

**Published:** 2025-10-08

**Authors:** Renato Elias Moreira Júnior, Mírian Velten Mendes, Mariana Siqueira Amormino, Gwenaëlle Randuineau, Célia Le Boulenger, Sylvie Guérin, Véronique Romé, Gaelle Boudry, Ana Lúcia Brunialti‐Godard

**Affiliations:** ^1^ Laboratório de Genética Animal e Humana, Departamento de Genética, Ecologia e Evolução Instituto de Ciências Biológicas–Universidade Federal de Minas Gerais Brazil; ^2^ Institut NuMeCan, INRAE, INSERM Univ Rennes France

**Keywords:** alcohol‐related disorders, cecal microbiota, dysbiosis, ethanol preference, gene expression regulation, obesogenic memory

## Abstract

Excessive consumption of high‐fat, high‐sugar diets promotes obesity, metabolic syndrome, and chronic inflammation through increased fat accumulation. While adopting a balanced diet promotes weight loss and improvements in various physiological parameters, persistent alterations in microbiota composition and function, metabolic imbalances, and behavioral changes may increase vulnerability to ethanol consumption and preference. In this context, the present study aims to investigate how switching from a high‐sugar, high‐saturated fat diet (HSB) to a standard diet (AIN93G) affects: (I) cecal microbiota composition and function, (II) colonic homeostasis, and (III) vulnerability to voluntary ethanol consumption in mice. To conduct the study, six animals were maintained on the standard diet, while 12 were given the HSB diet for 8 weeks. After this period, the HSB mice were switched to AIN93G for 4 weeks, with one subgroup given access exclusively to water (SWITCH) and another to water or a 10% ethanol solution (SWITCH+EtOH). The evaluation included measurements of body weight, adiposity index, cecal microbial composition, metabolomic profile, gut and hepatic morphology, transcriptional regulation of genes involved in colonic homeostasis and striatal dopaminergic neurotransmission, as well as ethanol consumption and preference. The results indicate that switching to the standard diet does not completely reverse obesity‐induced alterations. Persistent cecal dysbiosis, metabolic imbalances, and dopaminergic sensitization increase the predisposition to compulsive alcohol consumption and perpetuate epithelial and hepatic dysfunction. Therefore, post‐obesity interventions should combine weight management with strategies to restore microbiota and intestinal barrier function, along with measures to reduce vulnerability to reinforcement‐seeking behaviors.

## Graphical abstract

Male C57BL/6 mice were switched from a high‐sugar/high‐fat diet to a control diet with or without free‐choice 10% ethanol. The switch reshaped the cecal microbiota and metabolome (alpha diversity increased; polyamines and selected bile acids decreased) and reduced colonic barrier markers. Under free‐choice, ethanol preference over water was observed and impulsivity increased; cecal microbiota composition was altered; free amino acids and γ‐glutamyl derivatives decreased; cytosine increased; *Lyz* mRNA decreased while *Cldn7* mRNA increased; liver showed steatosis and inflammatory foci

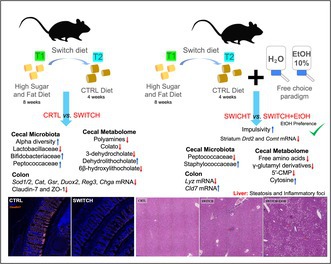

## 1 Introduction

Obesity is a leading cause of death and a significant risk factor for numerous health conditions [1, 2]. One of the main factors driving this condition today is the increasing consumption of hypercaloric diets rich in saturated fats and refined sugars [3, 4, 5]. This dietary pattern promotes excessive fat accumulation, leads to metabolic syndrome, and triggers low‐grade inflammatory dysfunctions that disrupt homeostasis [6, 7]. Additionally, obesity negatively affects self‐perception, which can contribute to anxiety and depression [8, 9, 10]. In response to these harmful effects, therapeutic strategies to improve metabolic health and related comorbidities have become a primary focus in obesity treatment [11, 12, 13]. One widely adopted approach is dietary re‐education, which involves modifying eating patterns and implementing caloric restriction, often leading to weight loss and improved metabolic parameters [12, 13, 14]. However, growing evidence suggests that restoring body weight does not guarantee the complete reversal of the alterations induced by obesity and the chronic intake of diets high in sugar and fat. This phenomenon is known as metabolic memory or obesogenic memory [14, 15, 16, 17].

Besides metabolic disturbances, obesity is also associated with altered eating behavior. Nutrients can affect neural reward and behavioral pathways by altering neurotransmitter levels, particularly dopamine [18, 19]. Experimental evidence indicates that prolonged intake of palatable foods can induce compulsive behavior and alter the transcriptional regulation of dopaminergic genes in the striatum, such as *Drd1*, *Drd2*, and *Slc6a3*, in a manner consistent with the activation of the mesolimbic reward system [18, 20, 21, 22]. The abrupt withdrawal of hedonic stimuli such as fatty and sweet food items to favor weight loss or adopting a balanced diet may sensitize dopaminergic circuits, promoting reinforcement‐seeking behaviors [20, 23, 24]. In humans, this phenomenon has been associated with relapse into maladaptive eating patterns or the substitution of compulsive food‐related behaviors with other forms of gratification, such as alcohol consumption [24, 25, 26, 27]. In post‐bariatric surgery populations, there is a significant increase in the prevalence of alcohol use disorders (AUD), which may be partially explained by neurobiological and behavioral adaptations induced by prior exposure to obesogenic diets [25, 26, 27, 28, 29]. Animal models support this hypothesis, showing that the withdrawal from hypercaloric diets can redirect compulsive behaviors toward ethanol intake, suggesting an overlap between the reinforcement systems activated by palatable foods and psychoactive substances [20, 23, 30]. Thus, individuals previously sensitized to dopaminergic reinforcement through high‐fat, high‐sugar diets may display greater vulnerability to alcohol abuse.

The exact molecular mechanisms behind metabolic memory and vulnerability to psychoactive substances following high‐fat and high‐sugar diet withdrawal remain largely unknown. However, this phenomenon has been described in several biological systems, including adipose tissue, hepatic metabolism, immune responses, and, increasingly, in the gut microbiota [14, 16, 17, 31, 32]. Obesogenic diets induce intestinal dysbiosis, marked by reduced microbial diversity, the loss of beneficial strains, lower production of regulatory metabolites such as short‐chain fatty acids (SCFA) and polyamines, and intestinal epithelial barrier impairment [33, 34, 35, 36]. These changes can persist even after restoring body weight and caloric intake, resulting in subclinical inflammation and residual metabolic dysfunctions [16, 37]. The involvement of the gut microbiota in food‐reward related behaviors through the gut‐brain axis has been progressively uncovered [38]. Gut microbiota dysregulation also seems to contribute to this alcohol use disorder vulnerability [30, 39, 40, 41]. Furthermore, metabolic and inflammatory alterations resulting from obesogenic feeding may further impair intestinal and hepatic function, exacerbating the deleterious effects of ethanol.

Given this context, the present study aimed to investigate the effects of transitioning from a high‐fat, high‐sugar diet to a standard diet on the composition and function of the cecal microbiota and colonic homeostasis. Additionally, it seeks to assess how these changes influence vulnerability to high ethanol intake and preference. The cecal content was selected for microbiota analysis because it exhibits lower variability compared to fecal microbiota, offering a more accurate representation of the direct interactions between microorganisms and the intestinal epithelium [42, 43]. A mouse model was employed, in which animals were exposed to a high‐sugar and butter (HSB) diet for 8 weeks, followed by 4 weeks on a standard diet. A subgroup of these mice was given voluntary access to a 10% ethanol (EtOH) solution to assess alcohol preference and consumption. Our hypothesis is that prior exposure to the HSB diet leads to lasting metabolic and neurobehavioral changes that promote compulsive alcohol‐seeking behavior. We believe these effects are supported by residual dysbiosis, transcriptional regulation alterations, and impaired intestinal function.

## 2 Materials and Methods

### 2.1 Animals

Eighteen six‐week‐old male C57BL/6 mice, Specific Pathogen‐Free, were obtained from the Animal Facility of the Universidade Federal de Minas Gerais (UFMG), Brazil. Under controlled environmental conditions, the animals were individually housed in mini‐isolators within a ventilated rack system (ALESCO, Brazil). Mice had ad libitum access to diet and drinking solutions throughout the study. All experimental procedures were approved by the Institutional Animal Care and Use Committee of UFMG (CEUA‐UFMG, protocol number 073/2021) and were performed following institutional guidelines to ensure animal welfare.

### 2.2 Experimental Design

**FIGURE 1:**
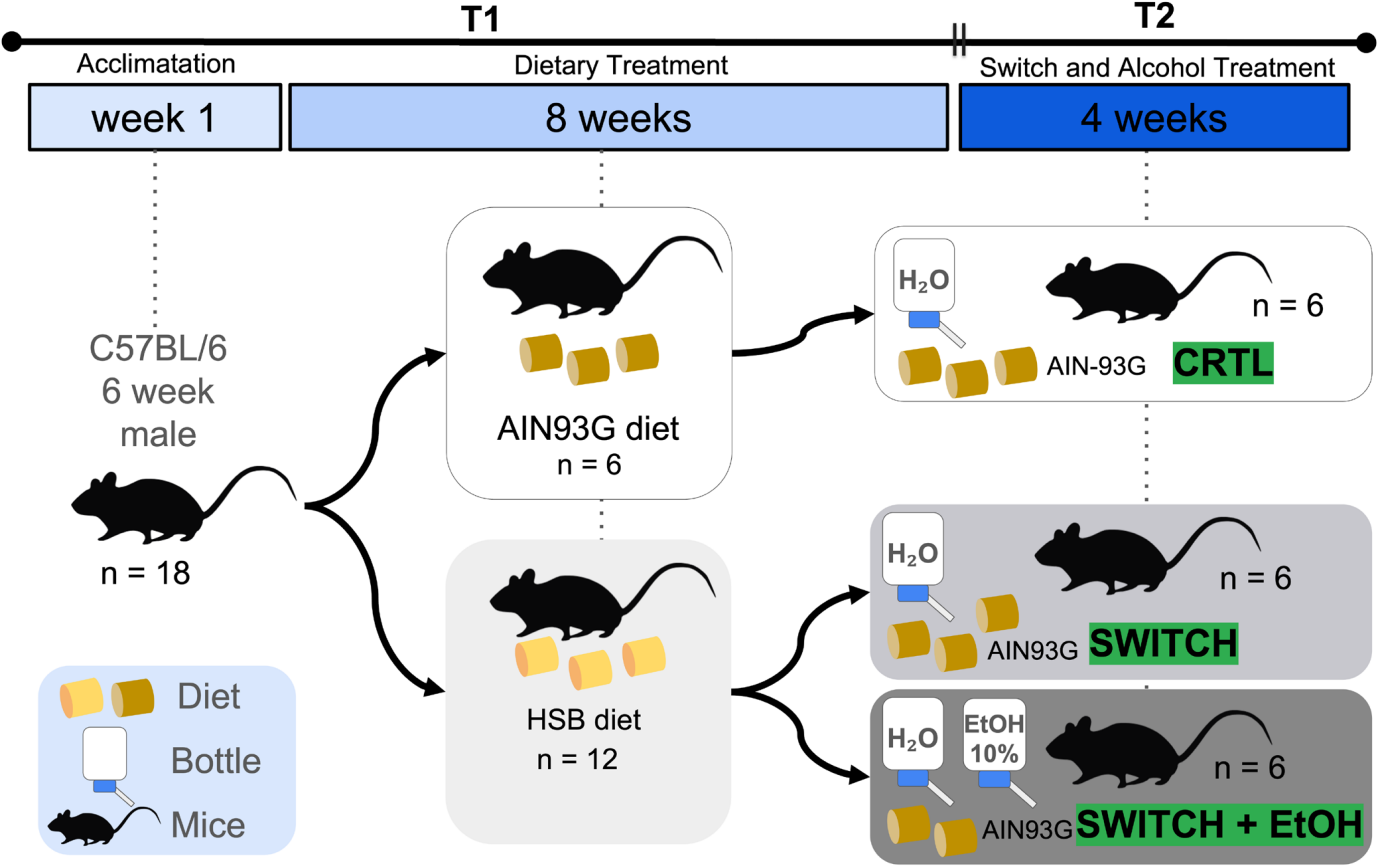
Experimental design. Following a 1‐week acclimatization period, 18 male mice were randomly assigned to three groups (*n* = 6). In Phase T1 (8 weeks), the control group received the AIN93G standard diet, while the experimental groups were fed a high‐sugar, high‐saturated fat diet (HSB). In Phase T2 (4 weeks), all animals were switched to the AIN93G diet; one subgroup had access to water only (SWITCH) and the other to both water and a 10% ethanol solution (SWITCH+EtOH). Body weight was recorded weekly, and fluid consumption was monitored daily during T2. All mice were euthanized at the end of T2, and samples were collected for further analysis.

The experimental design was adapted from Martins de Carvalho et al. [20]. After a one‐week acclimatization period, 18 male mice were randomly assigned to three groups (*n* = 6 per group). For 8 weeks (T1 phase), six animals received a standard diet formulated according to the American Institute of Nutrition 93‐Growth guidelines (AIN93G; carbohydrates: 64%, protein: 20%, fat: 16%; 3.9 kcal/g) and constituted the control group (CTRL), while 12 animals were fed a high‐sugar, high‐saturated fat diet (HSB; carbohydrates: 36%, protein: 16%, fat: 48%; 4.9 kcal/g) [35, 44]. Detailed formulations of the diets are provided in Table S1. Following this initial phase, animals in the experimental groups underwent a dietary switch to the standard AIN93G diet during a subsequent four‐week period (T2). At this stage, one subgroup had access only to water (SWITCH, *n* = 6) while the other was subjected to a two‐bottle choice ethanol paradigm (SWITCH+EtOH, *n* = 6), in which both water and a 10% ethanol solution were continuously available in the home cage. Body weight was recorded weekly throughout the experiment, and during T2, ethanol and water consumption were monitored daily. The experimental design is depicted in Figure 1.

On the final experimental day, animals were euthanized by anesthetic intraperitoneal injection, with death confirmed by cardiac arrest and absence of reflexes. Following euthanasia, the liver and perigonadal adipose tissue were excised and weighed. Under sterile conditions, cecal content was collected to analyze SCFAs, microbiome (16S rRNA gene sequencing), and metabolome profiling. Additionally, 1 cm of the proximal colon and the striatum were collected for transcriptional analysis of target genes. The remaining section of the colon and liver were preserved for histological evaluation. Tissues intended for molecular analyses were immediately snap‐frozen in liquid nitrogen and stored at −80 C, while samples destined for histological examination were promptly fixed in neutral‐buffered formalin.

### 2.3 Adiposity Index

After collection, the perigonadal adipose tissues were rinsed with saline solution and weighed. The adiposity index was calculated as the ratio between the perigonadal fat mass (in grams) and the animal's total body weight (in grams) [20].

### 2.4 Ethanol Consumption and Preference

Measurements were performed according to the method previously described [20, 30]. Daily intake of water and ethanol was determined by subtracting the final bottle weight from the initial weight (in grams) and normalizing to the animal's body weight for the corresponding week. The 10% ethanol solution was replaced after each measurement to minimize loss due to evaporation. Ethanol preference was considered significant when the percentage of ethanol solution consumed relative to total fluid intake was statistically greater than the hypothetical value of 50.1%.

### 2.5 Marble Burying Test

On the final day of T2, animals were subjected to the marble burying test, a widely used paradigm to assess impulsive and compulsive‐like behaviors in rodents [45, 46, 47]. The procedure and analysis were conducted as described by previously established protocols [20, 45]. Each animal was individually placed in an open plastic cage containing a 5 cm layer of woodchip bedding, with 18 glass marbles evenly arranged in three aligned rows. After a 10‐min exploration, the number of marbles buried at least two‐thirds beneath the bedding was recorded. Scoring was performed independently by two blinded investigators, and the average number of buried marbles per animal was used for statistical analysis.

### 2.6 Transcriptional Regulation of Genes in the Colon and Striatum

Total RNA was extracted from 1 cm‐segments of proximal colon using the NucleoSpin RNA Plus Kit (Macherey‐Nagel, Germany) and from striatal tissue using the ReliaPrep RNA Tissue Miniprep System (Promega, USA), following the manufacturers' protocols. For reverse transcription, colon and striatal RNA were converted to cDNA using the High‐Capacity cDNA Reverse Transcription Kit (Thermo Fisher Scientific) according to the manufacturer's protocol.

Quantitative PCR on colon samples was performed on a QuantStudio 7 Flex System with a 384‐well block using SYBR Green in technical triplicate. Cycling conditions consisted of an initial denaturation at 95°C for 2 min, followed by 40 cycles of 95°C for 15 s and 60°C for 60 s, with fluorescence acquisition at the end of each extension step. Striatal qPCR was carried out on a QIAquant.96 Real‐Time PCR System (QIAGEN) using SYBR Green under cycling conditions of 95°C for 2 min, then 40 cycles of 95°C for 5 s and 60°C for 10 s, also with real‐time fluorescence detection. All runs concluded with a melt curve analysis from 60°C to 95°C at 0.3°C/s to confirm single‐product amplification; no‐template controls showed no amplification, and standard deviations of Ct across triplicates were < 0.3. Primers used are listed in Table S2. *Gapdh* served as the reference gene for colon and striatum, *Hprt1* for the colon, and *Actb* for the striatum. Relative expression of target genes was calculated by the 2^−ΔΔCT^ method, normalizing each sample's ΔCt to that of the control group.

### 2.7 Cecal SCFA Determination

Cecal SCFA levels were determined using gas–liquid chromatography as previously described [48], with adjustments. Briefly, samples were subjected to water extraction followed by protein precipitation using phosphotungstic acid. The resulting supernatant was analyzed in duplicate on an Agilent Technologies 6890N system (Bios Analytique, Toulouse, France), equipped with a split injector (5:1) at 180°C and a flame ionization detector (FID) set at 180°C. Separation was performed on a bonded silica capillary column (30 m × 0.25 mm internal diameter; 0.25 μm film thickness; SolGel‐Wax; SGE, Villeneuve‐St‐Georges, France) with a polar stationary phase. Hydrogen was used as the carrier gas at an average velocity of 1.8 mL/min. The column temperature was programmed to start at 90°C and ramp at 5°C/min to 180°C. SCFAs were identified by comparing retention times to those of a commercial SCFA standard mix (Volatile Free Acid Mix CRM46975, Sigma Aldrich). Data acquisition and peak integration were performed using Agilent OpenLab ChemStation version B.04.03 SP1. A total of six SCFAs were identified and quantified: acetic, propionic, iso‐butyric, butyric, iso‐valeric, and valeric acids.

### 2.8 Cecal Microbiota Composition and Diversity Analysis

The cecal microbiota composition was determined by sequencing the V3–V4 region of the 16S rRNA gene. Total DNA was extracted from cecal content using the *Quick‐DNA Fecal/Soil Microbe Miniprep Kit* (Zymo Research, USA), following the manufacturer's instructions. DNA concentration and purity were assessed using a DeNovix spectrophotometer. For each sample, 12 ng of total DNA was diluted to a final volume of 15 μL, yielding a concentration of 0.8 ng/μL. PCR amplification was performed in a final volume of 60 μL, containing 5× Phusion HF Buffer, 0.4 μM of each primer (V3F: CTTTCCCTACACGACGCTCTTCCGATCTACTCCTACGGGAGGCAGCAG and V4R: GGAGTTCAGACGTGTGCTCTTCCGATCTTACCAGGGTATCTAATCC), 0.2 mM of dNTPs, 0.2 U of Phusion High‐Fidelity DNA Polymerase, and the diluted DNA template. The thermal cycling protocol consisted of an initial denaturation at 98°C for 30 s, followed by 25 cycles of 98°C for 10 s, 62°C for 30 s, and 72°C for 30 s, with a final extension at 72°C for 5 min. Electrophoresis on a 2% agarose gel using 1× TAE buffer at 100 V for 1 h confirmed amplification efficiency and specificity. Amplicons were submitted to the Genotoul GeT‐PlaGe platform (INRAE, Toulouse, France) for sequencing on an Illumina MiSeq system using paired‐end reads of 2 × 250 bp.

Sequencing data were processed using the FROGS pipeline version 4.1 [49]. Processing steps included primer and low‐quality read removal, OTU clustering via Swarm (aggregation distance *d* = 1), and chimera filtering. OTUs with relative abundances below 0.005% or present in fewer than two samples were excluded. Taxonomic assignment was performed against the SILVA 138.1 database with a pintail quality threshold of 100. The processed dataset was imported into a *phyloseq* object in R version 4.2.0 [50, 51]. Rarefaction to the minimum sequencing depth was applied before calculating alpha diversity indices, including observed richness and Simpson's index, and beta diversity based on Bray‐Curtis and Jaccard distances. Ordination methods, such as multidimensional scaling and relative abundance plots, were generated using the *phyloseq* and *vegan* R packages.

### 2.9 Cecal Metabolome

For metabolomic analysis, cecal content samples (~20–50 mg) were homogenized on ice with 200 μL of extraction solvent, incubated at 4°C for 15 min, and centrifuged at 14,000×*g* for 10 min. The resulting supernatant was transferred for UPLC–MS/MS injection. The Metabolon platform employed four chromatographic/ionization modes—positive HILIC (Polar), positive reversed‐phase early (Pos Early), positive reversed‐phase late (Pos Late), and negative reversed‐phase (Neg)‐acquiring both full‐scan and high‐resolution MS/MS fragmentation data. Peak areas were extracted using proprietary software and subjected to intra‐batch normalization to correct analytical drift, followed by imputation of missing values via empirical estimation and log_2_ transformation to stabilize the variance. Each detected feature was annotated by matching to an internal library and mapped to metabolic super‐pathways.

### 2.10 Liver Histology

Liver samples were immediately rinsed in physiological saline, placed in histological cassettes, and fixed in 10% neutral‐buffered formalin for 24 h. After fixation, tissues were transferred to 70% ethanol, dehydrated through graded alcohols, cleared in xylene, and embedded in paraffin. Sections of 4 μm thickness were cut, stained with hematoxylin and eosin (H&E), and digitized using a 3D HISTECH whole‐slide scanner (Hungary). Histopathological evaluation was performed independently by two observers blinded to experimental groups. Steatosis and lobular inflammation were semi‐quantitatively assessed according to established criteria [52, 53]. Steatosis was graded based on the proportion of hepatocytes containing lipid vacuoles per microscopic field at 20× magnification: grade 0, < 5%; grade 1, 5%–25%; grade 2, 26%–50%; grade 3, 51%–75%; and grade 4, > 75%. Inflammation was graded based on the number of inflammatory foci (clusters of infiltrating mononuclear cells) per area at 20× magnification: grade 0, no foci; grade 1, 1–2 foci per field; grade 2, 2–4 foci per field; and grade 3, more than 4 foci per field [53].

### 2.11 Colon Immunohistochemistry

Colonic samples were fixed for 24 h in 4% paraformaldehyde, then rinsed in 70% ethanol before being embedded in paraffin. Immunostaining was performed on 4‐μm sections. Slides were first deparaffinized. For immunostaining necessitating unmasking, slides were placed for 15 min in citrate buffer pH 6 (Immunoretriever 20X with citrate) for ZO‐1 or in EDTA buffer pH 9 (Immunoretriever 20X with EDTA) for Muc2, at 110°C and low pressure in a Tintoretrever‐Pressure‐Cooker (Diagomics, Blagnac, France). Slides were then rinsed with PBS for 15 min and blocked and permeabilized with a 0.25% Triton X‐100 (Merck T8787, Darmstadt, Germany)‐3% horse serum (Merck, ref. H1270) PBS solution for 30 min. The slides were rinsed with PBS, then 50 μL of primary antibody (Claudin 7: ab27487 (Abcam) 1/100 1 h; ZO‐1: 61‐7300 (Invitrogen) 1/50 overnight; Muc2: orb372331 (Biorbyt), 1/200, 1 h; chromogranin A: ab45179 (Abcam) 1/400 20 min) was deposited per section. After PBS rinse, 50 μL of secondary antibody (claudin 7: anti‐rabbit IgG Alexa Fluor 555 conjugate, 4413S (Ozyme), 1/500 1 h; ZO‐1: anti‐rabbit IgG Alexa Fluor 488 conjugate, 4412S (Ozyme), 1/250 1 h; Muc2: anti‐rabbit IgG Alexa Fluor 555 conjugate, 4413S (Ozyme), 1/250 1 h; ChgA: anti‐rabbit IgG Alexa Fluor 488 conjugate, 4412S (Ozyme) 1/500 30 min) were deposited at room temperature. Finally, the sections were rinsed for 15 min in PBS and then mounted under a coverslip using a mounting medium with DAPI (Aqueous Fluoroshield, Abcam104139, Cambridge, UK). Images were obtained using a Zeiss microscope Axio Imager M2 with an ApoTome 2 module. The objective lens used was 20×. Images were analyzed using ImageJ software. For Claudin 7 and ZO‐1, the intensity of the staining was scored from 0 to 3. For Muc2 and ChgA, the density of immunoreactive cells/mm^2^ was calculated. Crypt depth was measured on a minimum of 10 well‐oriented crypts on H&E stained sections. Operators were blinded to the experimental group.

### 2.12 Statistical Analyses

Statistical analyses were performed using GraphPad Prism software version 10 and R software. Data normality was assessed using the Shapiro–Wilk and Kolmogorov–Smirnov tests. Two‐tailed unpaired or paired Student's *t*‐tests were applied for parametric data, while non‐parametric data were analyzed using the Mann–Whitney or Kruskal–Wallis tests, as appropriate. Group comparisons involving two or more factors were evaluated using one‐way or two‐way ANOVA, followed by Tukey's or Sidak's post hoc tests. Two‐way ANOVA with repeated measures was employed for repeated measures. Microbiome data were explored with PCoA and nMDS based on Bray–Curtis or UniFrac distances, and differential abundance was assessed with DESeq2. Metabolomics data were summarized with Partial least squares discriminant analysis (PLS‐DA) and heatmaps. PLS‐DA score plots display the first two latent variables (LV1 and LV2) and were used to visualize overall patterns and group separation. For heatmaps, metabolite intensities were transformed to per‐metabolite row‐wise *z*‐scores and included all detected metabolites annotated by the platform within the Amino Acid super‐pathway and the γ‐glutamyl Amino Acid sub‐pathway. Ethanol preference was evaluated using a one‐sample *t*‐test comparing mean preference to a hypothetical 50.1% threshold. Results are presented as mean ± SEM for bar graphs and as minimum‐to‐maximum values for boxplots. Statistical significance was defined as *p* < 0.05.

## 3 Results

### 3.1 Dietary Switch Modulates Body Weight and Cecal Microbiota Composition

**FIGURE 2:**
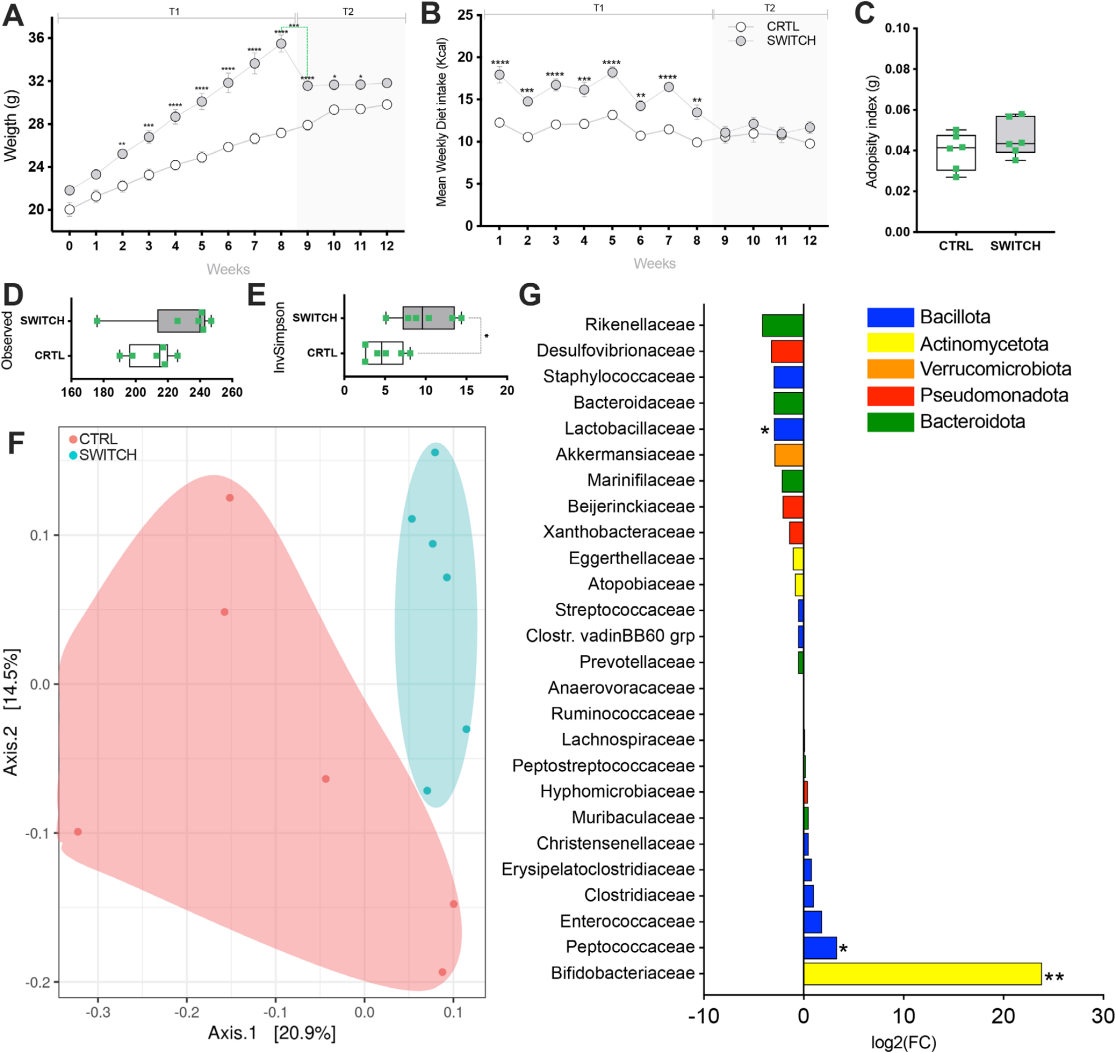
Dietary switch alters the structure and diversity of the mouse cecal microbiota. Mice were initially divided into two groups: CTRL (*n* = 6), which received a control diet (AIN93G) for 12 weeks, and SWITCH (*n* = 6), which received a high‐sugar and butter diet (HSB) for 8 weeks, followed by a switch to the AIN93G diet for the remaining 4 weeks. (A) Body weight progression throughout the experiment. (B) Mean Weekly Diet Intake (kcal). (C) Adiposity index at the end of the experiment. (D) Microbial richness (number of observed OTUs). (E) Microbial alpha diversity measured by the Inverse Simpson index. (F) Non‐metric multidimensional scaling representation of the microbiota structure based on the UniFrac distance. (G) Differential abundance analysis at the family level between groups. Bars are colored according to their corresponding phylum. Statistical analysis: (A, B) Two‐way repeated measures ANOVA followed by Sidak's post hoc test for comparisons between groups over time. Paired *t*‐test was used to assess within‐group differences between Week 8 and 9 (green dashed line). (C–E) Unpaired two‐tailed *t*‐tests (F) PERMANOVA based on PCoA from UniFrac distance matrix (G) Differential abundance was assessed using DESeq2 analysis. Box plots show minimum to maximum values, with individual data points overlaid. *p < 0.05, **p < 0.01, ***p < 0.001, ****p < 0.0001.

Mice in the SWITCH group exhibited significantly higher body weight compared to the CTRL group throughout the 8 weeks of the HSB feeding phase (T1), with a reduction in weight gain observed immediately after the dietary switch between Weeks 8 and 9 (*p* = 0.0009). During T2, body weight remained elevated in the SWITCH group at Weeks 9, 10, and 11 (Figure 2A). There were no differences in food consumption (in grams) between the groups over the weeks. However, considering caloric intake during the 8 weeks of T1, animals in the group fed the HSB diet showed higher energy intake. In contrast, no significant differences were observed during T2 (Figure 2B). Consistent with body weight at Week 12, the adiposity index was also not significantly different between the CTRL and SWITCH groups (*p* = 0.2427; Figure 2C).

Although no significant differences in microbial richness were observed between groups (*p* = 0.1677; Figure 2D), alpha diversity was significantly higher in the SWITCH group (*p* = 0.0137; Figure 2E). nMDS analysis based on UniFrac distances revealed a clear separation in cecal microbial community composition between CTRL and SWITCH groups (Figure 2F). No differences were observed at the phylum level between groups. However, differential abundance analysis at the family level identified several taxa significantly altered by the dietary intervention, including reduced abundance of Lactobacillaceae and increased abundance of Bifidobacteriaceae and Peptococcaceae in the SWITCH group (Figure 2G).

### 3.2 Dietary Switch Alters the Cecal Metabolome

**FIGURE 3:**
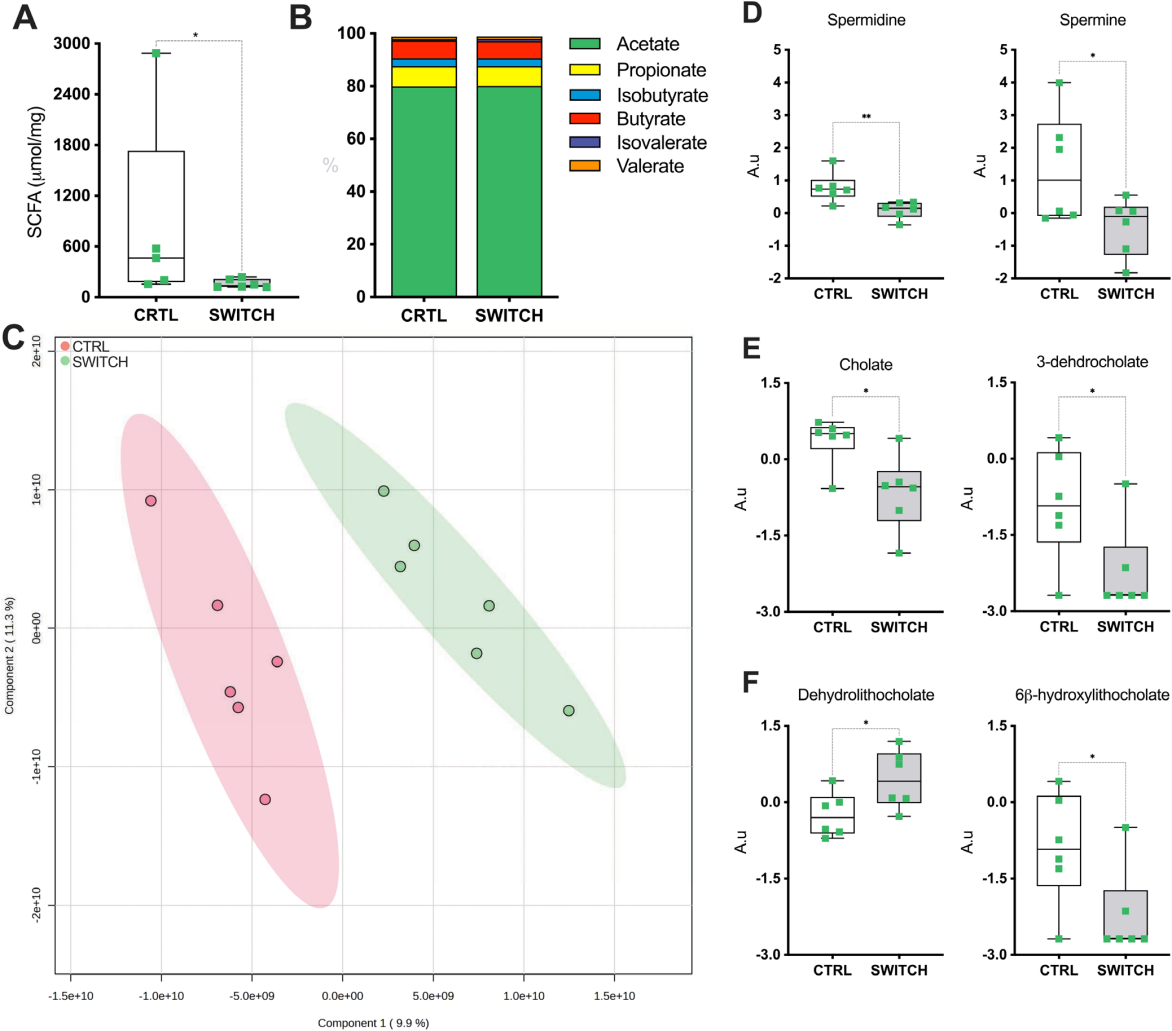
The dietary switch impacted the mouse cecal metabolome. (A) Total cecal short‐chain fatty acid (SCFA) concentrations. (B) Relative proportions of individual SCFAs. (C) PLS‐DA of the cecal metabolomic profile, score plot of the first two latent variables (LV1 and LV2). (D) Relative concentrations of bioamines. (E) Relative concentrations of primary bile acids and derivatives. (F) Relative concentrations of secondary bile acids. Dots represent individual values. Statistical analysis: (A, D–F) were analyzed using unpaired two‐tailed *t*‐tests. (C) was based on PLS‐DA visualization. Box plots show minimum to maximum values, with individual data points overlaid. *p < 0.05, **p < 0.01.

Total concentrations of SCFAs in the cecal content differ significantly between groups (*p* < 0.05), but the relative proportions of individual SCFAs remained relatively consistent (Figure 3A,B). Moreover, PLS‐DA analysis revealed a distinct separation in cecal metabolomic profiles between CTRL and SWITCH groups, indicating a broad shift in cecal metabolite composition induced by the dietary intervention (Figure 3C). In this context, among the bioamines analyzed, the SWITCH group exhibited significantly lower levels of spermidine (*p* = 0.0087) and spermine (*p* = 0.0462) compared to CTRL (Figure 3D). Primary and secondary bile acids were also affected by the dietary switch. Among the primary bile acids, cholate (*p* = 0.0260) and its derivative 3‐dehydrocholate (*p* = 0.0428) were significantly reduced in the SWITCH group (Figure 3E). Regarding secondary bile acids, dehydrolithocholate levels were significantly elevated (*p* = 0.0392), whereas 6β‐hydroxylithocholate was reduced (*p* = 0.0428) in the SWITCH group (Figure 3F).

### 3.3 Dietary Switch Disrupts Colonic Epithelial Homeostasis

**FIGURE 4:**
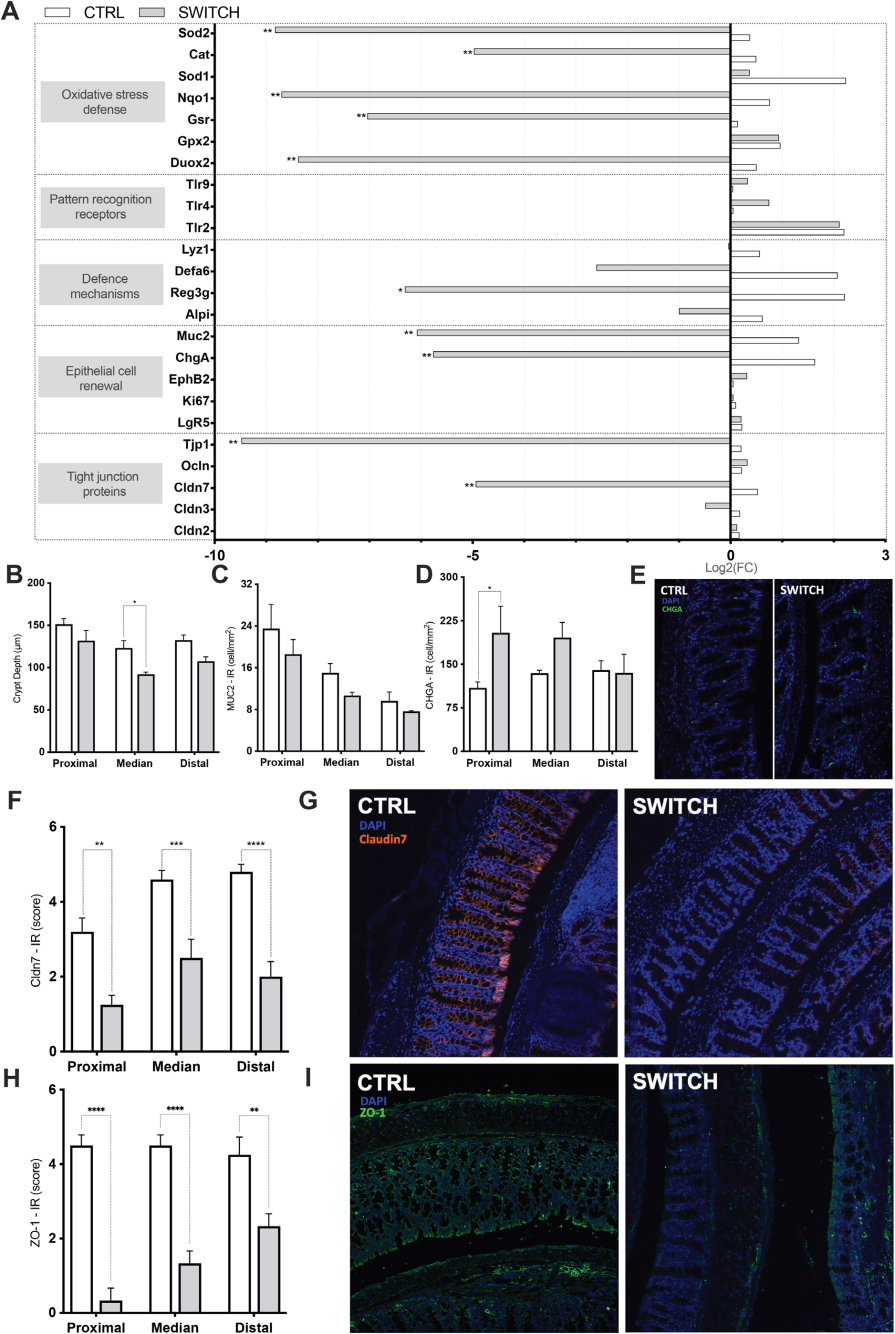
The dietary switch impacted mouse colonic epithelium homeostasis. (A) Relative gene expression (log_2_ fold change) of markers related to colonic homeostasis. (B) Colonic crypt depth in proximal, median, and distal segments. (C) Colonic MUC2 immunoreactive cell density. (D) Colonic chromogranin A (CHGA) immunoreactive cell density. (E) Representative images of CHGA staining (green) and DAPI nuclear staining (blue) in the median colon (F) Claudin 7 immunoreactivity score across colonic segments. (G) Representative images of claudin 7 staining (red) and DAPI nuclear staining (blue) in the distal colon of CTRL and SWITCH groups. (H) ZO‐1 immunoreactivity score across colonic segments. (I) Representative images of ZO‐1 staining (green) and DAPI nuclear staining (blue) in the distal colon of CTRL and SWITCH groups. Statistical analysis: (A) analyzed by unpaired two‐tailed *t*‐tests; (B–E, G) by two‐way ANOVA followed by Tukey's post hoc test. Bars represent mean ± SEM. *p < 0.05, **p < 0.01, ***p < 0.001, ****p < 0.0001.

The transcriptional profile in the proximal colon of SWITCH animals was altered, with several markers associated with epithelial homeostasis being significantly downregulated in the SWITCH group (Figure 4A). These included genes involved in oxidative stress defense (*Sod2*, *Cat*, *Sod1*, *Gsr*, *Duox2*), antimicrobial defense (*Reg3g*), epithelial defense (*Muc2*), and endocrine function (*Chga*) and tight junction integrity (*Tjp1*, *Cldn7*).

Crypt depth was significantly reduced in the median colon of SWITCH animals compared to CTRL (*p* = 0.0367), while no differences were observed in the proximal and distal segments (Figure 4B). MUC2‐immunoreactive cell density showed a decreasing trend along the colon; however, no significant differences were found between groups in any segment (Figure 4C). In contrast, the number of chromogranin A (CHGA)‐positive cells was significantly higher in the proximal colon of SWITCH mice (*p* = 0.0317; Figure 4D). Claudin 7 and ZO‐1 immunoreactivity were significantly reduced (*p* < 0.05) across all colonic segments in SWITCH animals compared to CTRL (Figure 4F,H). Representative images of the distal colon confirm reduced Claudin 7 and ZO‐1 signals in the SWITCH group (Figure 4G,I).

### 3.4 Ethanol Free‐Choice Following Dietary Switch Promotes High Ethanol Intake and Preference

**FIGURE 5:**
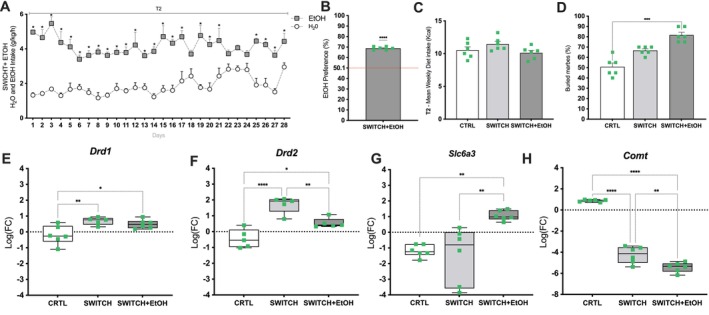
Dietary switch leads to high ethanol intake and preference in the free‐choice drinking paradigm. (A) EtOH and water intake (g/kg/h) over 28 days in the SWITCH+EtOH group. (B) Ethanol preference (%) in the SWITCH+EtOH group. (C) T2 Mean Weekly Diet Intake (kcal). (D) Buried marbles (%). (E) *Drd1* mRNA levels. (F) *Drd2* mRNA levels. (G) *Slc6a3* mRNA levels. (H) *Comt* mRNA levels. Statistical analysis: (A) Two‐way repeated measures ANOVA followed by Sidak's post hoc test; (B) One‐sample t‐test comparing ethanol preference to the hypothetical value of 50.1%; (C–H) One‐way ANOVA test. Bars represent mean ± SEM. Box plots show minimum to maximum values, with individual data points overlaid. *p < 0.05, **p < 0.01, ***p < 0.001, ****p < 0.0001.

During the T2 period, animals in the SWITCH group exposed to the ethanol free‐choice paradigm exhibited significantly higher ethanol intake (*p* < 0.05) compared to water across the days (Figure 5A). When animals from the CTRL group were subjected to the same paradigm, they did not exhibit the same behavior, instead showing an EtOH aversion (see Figure S1). Previous studies found no significant differences between CTRL+EtOH and CTRL groups in body weight, adiposity, behavior, metabolism, or microbiota composition [20, 23, 30]. Regarding body weight and adiposity index, SWITCH+EtOH animals showed no significant differences (*p* > 0.05) compared to the other groups. No differences were observed in dietary kilocalorie intake among the groups at T2 (Figure 5C). Moreover, animals in the SWITCH+EtOH group showed a strong ethanol preference (*t* = 29.18, df = 5, *p* < 0.0001), with the average intake significantly exceeding the hypothetical 50.1% threshold (Figure 5B). Ethanol consumption remained consistently elevated across the observation period, indicating a sustained preference for ethanol over water. In addition, the marble burying test revealed an increase in impulsivity‐like behavior, as animals in the SWITCH+EtOH group buried a significantly higher percentage of marbles (*p* = 0.0004) compared to the control group (Figure 5D).

Regarding transcriptional regulation in the striatum, *Drd1* transcription was significantly increased in both SWITCH (*p* = 0.0089) and SWITCH+EtOH (*p* = 0.0302) groups compared to CTRL (Figure 5E). *Drd2* transcription was elevated in the SWITCH+EtOH group (*p* = 0.0189) relative to CTRL but reduced compared to SWITCH (*p* = 0.0189) (Figure 5F). *Slc6a3* transcription was also higher in SWITCH+EtOH animals than in both CTRL (*p* = 0.066) and SWITCH (*p* = 0.027) groups (Figure 5G). In contrast, *Comt* transcription was reduced in SWITCH+EtOH animals compared to both CTRL (*p* < 0.0001) and SWITCH (*p* = 0.046) (Figure 5H).

### 3.5 Ethanol Consumption Leads to Increased Hepatic Fat Accumulation and Inflammatory Foci

**FIGURE 6:**
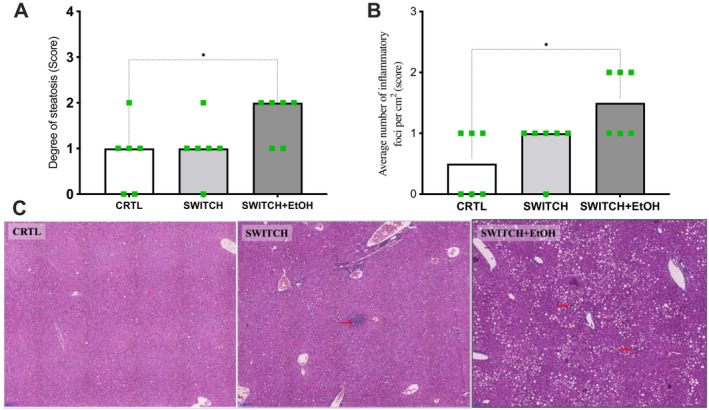
Ethanol consumption alters liver histology. (A) Hepatic steatosis score. (B) Inflammatory foci score (number of infiltrates per cm^2^). (C) Representative images of H&E‐stained liver sections at 20× magnification. The red arrow indicates an inflammatory foci. Statistical analysis: (A, B) were analyzed using the Kruskal–Wallis test. Bars represent the median, and dots represent individual values within the scoring criteria. *p < 0.05.

Animals from the SWITCH+EtOH group exhibited a higher percentage of fat‐accumulated hepatocytes (*p* = 0.0413) and a more significant number of inflammatory foci (*p* = 0.0208) compared to the CTRL group. According to the histological scoring criteria, these alterations indicate moderate hepatic steatosis and mild to moderate lobular inflammation (Figure 6).

### 3.6 High Ethanol Intake Selectively Modulates Cecal Microbiota Composition

**FIGURE 7:**
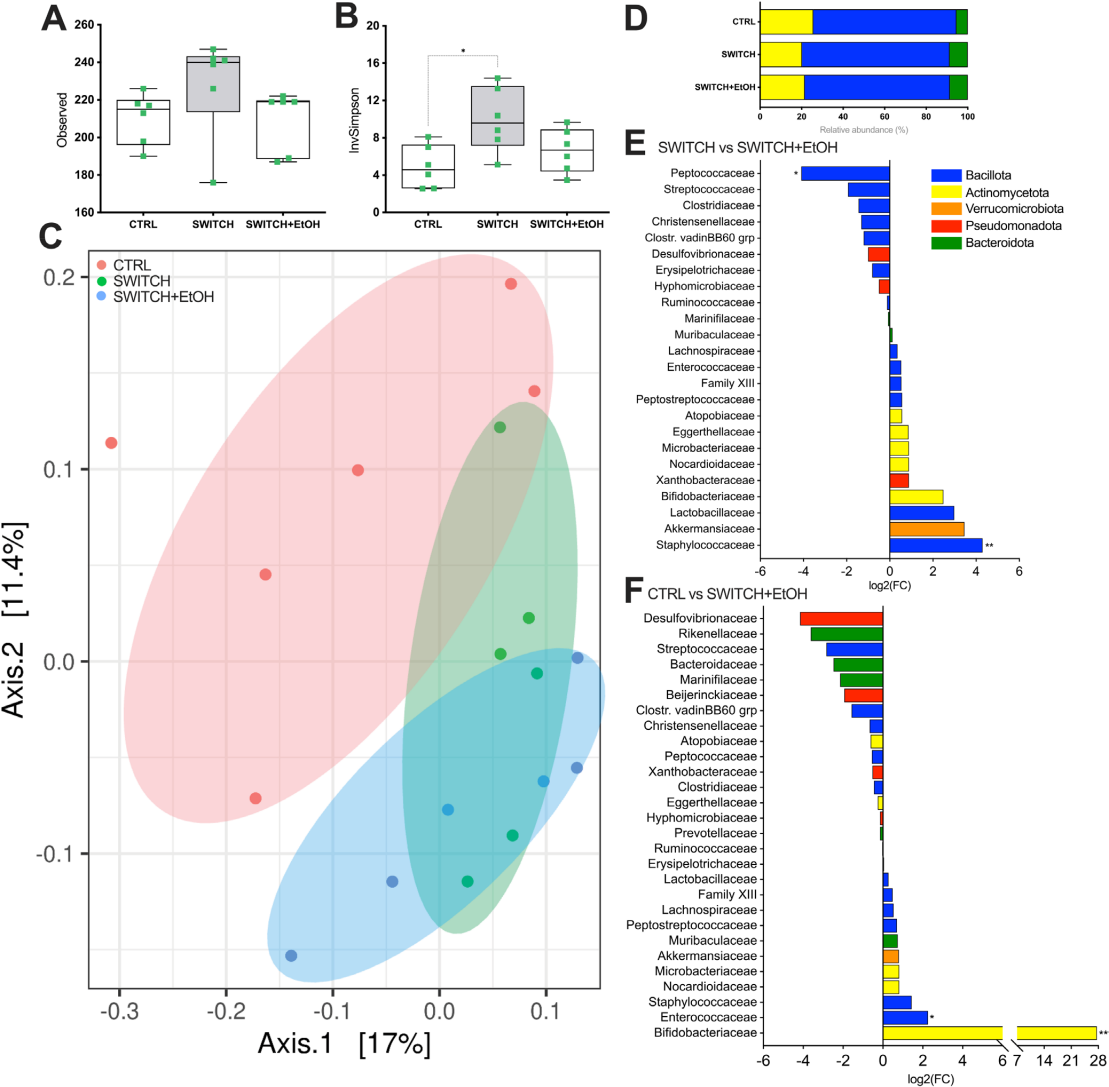
Ethanol exposure alters cecal microbiota composition. (A) Microbial richness (number of observed OTUs). (B) Microbial alpha diversity measured by the Inverse Simpson index. (C) Beta diversity plot (PCoA) based on the Bray–Curtis dissimilarity showing clustering by treatment group. (D) Relative abundance of major bacterial phyla in cecal microbiota. (E) Differentially abundant bacterial families between SWITCH and SWITCH+EtOH groups, displayed as log_2_ fold change. (F) Differentially abundant bacterial families between CTRL and SWITCH+EtOH groups, displayed as log_2_ fold change. Statistical analysis: (A, B) were analyzed using one‐way ANOVA followed by Tukey's post hoc test. (C) PERMANOVA based on PCoA from UniFrac distance matrix. (E, F) Differential abundance was assessed using DESeq2. Box plots show minimum to maximum values, with individual data points overlaid. *p < 0.05, **p < 0.01, ***p < 0.001, ****p < 0.0001.

High ethanol intake and preference did not significantly alter cecal microbiota richness, overall diversity, or phylum‐level composition in the SWITCH+EtOH group compared to the SWITCH group (Figure 7A–D). However, compared to the SWITCH group, a reduction in members of the Peptococcaceae family and an increase in Staphylococcaceae were observed (Figure 7E). Additionally, relative to the CTRL group, the SWITCH+EtOH group showed an increased abundance of Enterococcaceae and Bifidobacteriaceae families (Figure 7F).

### 3.7 SWITCH+EtOH Mice Exhibit Altered Cecal Amino Acid, SCFA, and Bile Acid Metabolism

**FIGURE 8:**
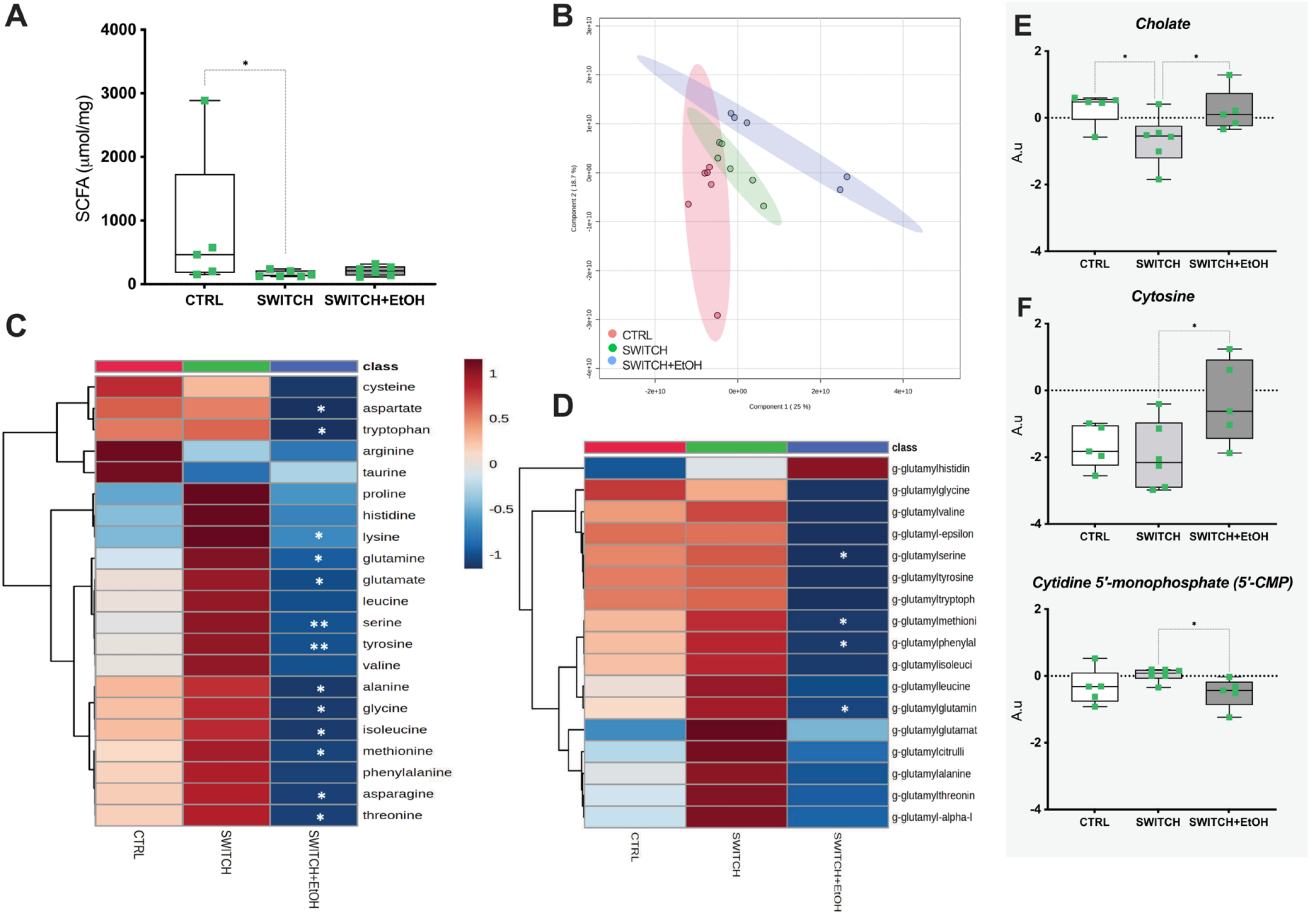
SWITCH + EtOH mice display a distinct cecal metabolomic profile compared to CTRL and SWITCH groups. (A) Total concentration of SCFAs in cecal content. (B) PLS‐DA showing separation of cecal metabolomic profiles by group, score plot of the first two latent variables (LV1 and LV2). (C) Heatmap of relative concentrations of amino acids including all detected metabolites. (D) Heatmap of relative concentrations of γ‐glutamyl amino acid derivatives including all detected metabolites. (E) Relative concentrations of Cholate. (F) Relative concentrations of selected nucleotides. Statistical analysis: (A) Kruskal–Wallis test. (B) was based on PLS‐DA visualization. (C, D) were analyzed using hierarchical clustering of z‐score normalized values. (E, F) were analyzed using one‐way ANOVA followed by Tukey's post hoc test. Box plots show minimum to maximum values, with individual data points overlaid. *p < 0.05, **p < 0.01.

The SWITCH+EtOH group did not exhibit significant differences in total SCFA concentrations compared to the other groups (Figure 8A). However, metabolic profiling revealed a distinct cecal profile that diverged from the CTRL and SWITCH groups. Multivariate analysis (PLS‐DA) demonstrated a clear separation among groups, indicating significant alterations in the cecal metabolome induced by ethanol exposure combined with dietary switch after timepoint T1 (Figure 8B).

In this context, a significant reduction was observed in the levels of several free amino acids, including aspartate, tryptophan, lysine, glutamine, glutamate, serine, tyrosine, alanine, glycine, isoleucine, methionine, asparagine, and threonine in SWITCH+EtOH versus SWITCH mice (Figure 8C). Moreover, animals in the SWITCH+EtOH group showed consistent reductions in γ‐glutamyl amino acid derivatives, particularly γ‐glutamyl‐serine, γ‐glutamyl‐methionine, γ‐glutamyl‐phenylalanine, and γ‐glutamyl‐glutamine compared to SWITCH mice (Figure 8D). Regarding bile acid metabolism, a trend for an increase (*p* = 0.0505) in the primary bile acid cholate was detected in the SWITCH+EtOH group compared to SWITCH, whereas cholate was significantly decreased (*p* = 0.0279) when comparing SWITCH and CTRL (Figure 8E). Hyocholate (*p* = 0.0651) and isohyodeoxycholate (*p* = 0.0609) tended to be reduced in SWITCH+EtOH compared to SWITCH. Finally, a significant increase in cytosine levels (*p* = 0.0476) and a decrease in cytidine 5′‐monophosphate (*p* = 0.0493) were found in the SWITCH+EtOH group when compared to SWITCH (Figure 8F).

### 3.8 SWITCH+EtOH Mice Exhibit Transcriptional and Morphological Changes in the Colon

**FIGURE 9:**
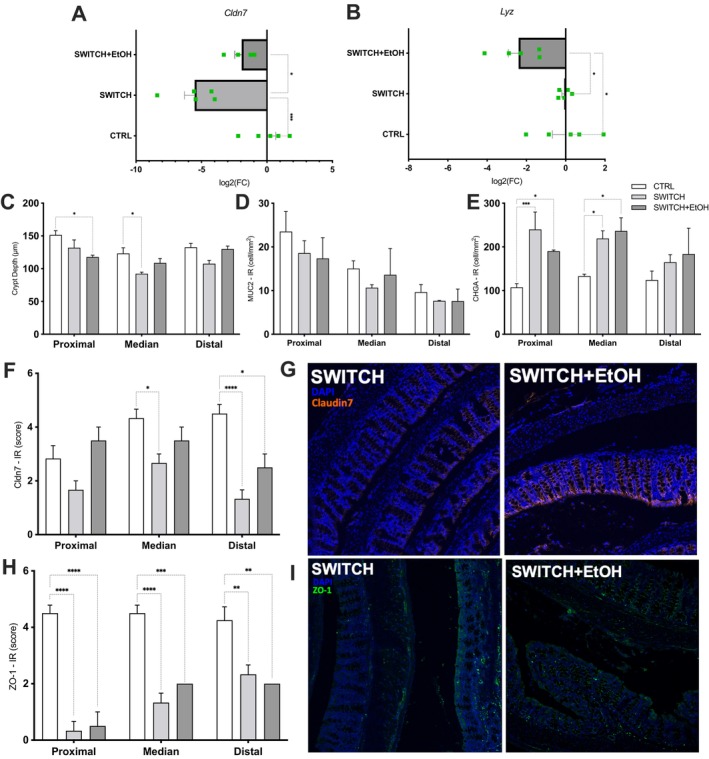
Colonic epithelial homeostasis is disrupted in SWITCH+EtOH mice. (A) Claudin 7 mRNA relative levels. (B) Lysozyme mRNA relative levels. (C) Crypt depth. (D) Colonic MUC2 immunoreactive cell density. (E) Colonic chromogranin A immunoreactive cell density. (F) Colonic Claudin 7 immunoreactivity score. (G) Representative images of Claudin 7 staining (red) in the distal colon; nuclei stained with DAPI (blue). (H) Colonic ZO‐1 immunoreactivity score. (I) Representative images of ZO‐1 staining (green) in the distal colon; nuclei stained with DAPI (blue). Statistical analysis: (A, B) were analyzed using one‐way ANOVA followed by Tukey's post hoc test; (C–F, H) were analyzed using two‐way ANOVA followed by Tukey's post hoc test. Bars represent mean ± SEM. *p < 0.05, **p < 0.01, ***p < 0.001, ****p < 0.0001.

No major differences in colonic gene expression were observed between SWITCH+EtOH and SWITCH mice. However, in the SWITCH+EtOH group, a significant increase in *Cldn*7 gene transcript levels was observed in the colon (*p* = 0.0109) compared to the SWITCH group, which, in turn, showed a significant reduction relative to the CTRL group (*p* = 0.0003), as demonstrated by the log_2_ fold change analysis (Figure 9A). Additionally, there was a downregulation of the *Lyz* gene, which encodes lysozyme, in the SWITCH+EtOH group compared to both the SWITCH (*p* = 0.0161) and CTRL (*p* = 0.0135) groups (Figure 9B).

Regarding colonic morphology, mice in the SWITCH+EtOH group showed a significant reduction in crypt depth in the proximal colon compared to the CTRL group (*p* = 0.0278) (Figure 9C). Although the density of MUC2‐immunoreactive cells did not differ significantly among groups, a regional gradient was observed, with decreasing density from proximal to distal segments (Figure 9D). Additionally, the density of CHGA‐immunoreactive cells was increased in the SWITCH+EtOH group compared to CTRL in both the proximal (*p* = 0.0441) and median (*p* = 0.0107) colon, with no significant differences observed between SWITCH and SWITCH+EtOH groups (Figure 9E). For Claudin 7 and ZO‐1, no differences were observed between SWITCH and SWITCH+EtOH. However, Claudin 7 staining was reduced in both SWITCH (*p* < 0.0001) and SWITCH+EtOH (*p* = 0.0209) groups compared to CTRL in the distal colon, and in the SWITCH group compared to CTRL (*p* = 0.0267) in the median colon. In contrast, ZO‐1 staining was significantly reduced in all colonic segments in both SWITCH and SWITCH+EtOH groups compared to CTRL (Figure 9F,H). Representative immunofluorescence images support these findings, showing reduced Claudin 7 (red) and ZO‐1 (green) staining in the SWITCH+EtOH group relative to CTRL, particularly in the distal colonic mucosa (Figure 9G,I).

## 4 Discussion

This study was designed to simulate a scenario frequently observed in human populations, characterized by prolonged consumption of high‐sugar and saturated‐fat diets, progressively leading to weight gain, increased body fat accumulation, and the development of metabolic changes typical of obesity [3, 54]. In response to the adverse effects of this dietary pattern, an abrupt shift in eating habits is commonly proposed, associated with a period of nutritional reeducation with a balanced diet [6, 11, 54]. To experimentally investigate this context, mice were fed an HSB diet for 8 weeks, during which they showed significant increases in body weight. Using the same highly palatable diet employed here, we previously reported in mice increased adiposity, metabolic dysregulation, and behavioral and gut microbiota alterations consistent with an obesogenic profile, as well as the diet's capacity to induce binge‐like eating under intermittent access [18, 20, 34, 35]. After replacing the HSB diet with a standard diet during the final 4 weeks of the study, animals exhibited a rapid reduction in body weight and adiposity. By the end of the experiment, these parameters did not differ significantly from those observed in the control group. In humans, similar interventions have been associated with improved health parameters, including weight loss, enhanced insulin sensitivity, and improved lipid profiles [3, 11]. However, metabolic and epigenetic changes triggered by obesity and diets rich in fats and sugars may persist, resulting in the phenomenon known as metabolic memory [6, 12, 15, 37, 55].

In SWITCH mice, cecal bacterial richness was unchanged, but α‐diversity increased, and β‐diversity remained distinct from that of CTRL mice, indicating community restructuring. A reduction in the relative abundance of the Lactobacillaceae family was observed, while the relative abundance of Bifidobacteriaceae and Peptococcaceae increased following the dietary shift. Although only a few bacterial families remained altered, functional differences emerged, supporting the concept of obesogenic memory [31, 37]. For example, the decreased abundance of Lactobacillaceae may imply reduced production of SCFAs, which are metabolites that nourish enterocytes and support immune responses through regulatory T cells [56, 57, 58, 59]. This could contribute to downregulating genes related to colonic epithelial defense and tight junction integrity, even after body weight recovery, reflecting persistent low‐grade inflammation induced by the previous obesogenic diet [17, 60, 61]. Indeed, Torres et al. demonstrated that chronic consumption of the HSB diet led to increased intestinal permeability, a reduction in the frequency of regulatory T cells in the lamina propria, and mucosal immune dysregulation, as reflected by a higher proportion of fecal bacteria coated with IgA [62]. The increased abundance of Peptococcaceae has been associated with metabolic disorders, where its enrichment may reflect enhanced proteolytic fermentation and contribute to intestinal dysbiosis and systemic inflammation [63, 64, 65]. SWITCH mice also exhibited an increase in Bifidobacteriaceae, whose members ferment complex carbohydrates through the bifid shunt, releasing lactic acid and acetate, an expansion that often signals a gut microbiota actively reassembling after disturbance [66, 67]. However, its impact can be host‐dependent [68]. Although still incomplete, this reassembly indicates residual dysbiosis, as the microbiota of animals that lose weight remains functionally distinct, likely retaining signatures of the obesogenic period and negatively influencing both epithelial regeneration and metabolic signaling.

The metabolomic profiles of cecal content revealed a significant decrease in spermidine and spermine, polyamines synthesized both by the intestinal epithelium and by commensal microorganisms [33, 69, 70]. These compounds support intestinal stem cell proliferation, the maintenance of tight junctions, and the integrated activation of the AhR, Nrf2, and STAT3 pathways, which coordinate the production of anti‐inflammatory cytokines and antioxidant defense mechanisms [33, 69, 71]. The reduction in polyamines is compatible with a loss of producer strains and/or decreased host production, which may, in turn, impair mucosal repair following metabolic or inflammatory insults [33, 71]. Concomitantly, a decrease in primary bile acids like cholate, 3‐dehydrocholate, and 6β‐hydroxylithocholate was observed, along with an increase in dehydrolithocholate, a hydrophobic secondary metabolite generated via 7‐ or 3‐dehydroxylase activity from bacteria [72, 73]. Given the cytotoxic properties of dehydrolithocholate, these findings suggest a predominance of microbial dehydroxylation pathways that may perpetuate epithelial damage and residual inflammation [72, 74, 75]. Furthermore, the marked depletion of glycine and serine, two amino acids required for synthesizing and stabilizing scaffold proteins such as ZO‐1 and Claudin‐7, suggests that substrate shortage may delay tight junction renewal, particularly in distal regions of the colon [76, 77].

Clinical and experimental evidence suggests that obesogenic memory and lingering physiological alterations after diet‐induced weight loss hinder the establishment of healthy eating habits [14, 15, 16, 37]. Abrupt withdrawal from hypercaloric diets sensitizes mesolimbic circuits, promoting the search for dopaminergic stimuli like ethanol [18, 25, 78, 79]. Moreira Júnior et al. showed that intermittent HSB diet induces compulsive‐like eating via transcriptional changes in mesolimbic dopaminergic genes [18]. Cohort studies consistently report high post‐bariatric AUD rates [25, 28, 29, 80, 81]. Preclinical models show that withdrawal from palatable diets increases ethanol intake, with sucrose‐conditioned or diet‐switched mice escalating alcohol consumption [20, 23, 82]. Ethanol activates the sweet receptor T1R3, whose absence abolishes ethanol preference [79, 83, 84, 85]. Together, these findings suggest that mesolimbic neuroadaptations and residual metabolic disturbances increase vulnerability to alcohol abuse. In this study, SWITCH mice showed greater ethanol intake and preference, and elevated marble burying, indicating enhanced compulsive‐anxious behavior [46, 47]. Increased *Drd1* and *Drd2* transcription, and ethanol‐induced *Slc6a3* upregulation and *Comt* downregulation suggest sustained dopaminergic signaling, consistent with compulsive reward, such as ethanol seeking [20, 21, 23, 86, 87]. Although voluntary ethanol consumption did not alter cecal microbiota diversity or phylum‐level distribution, discrete shifts in specific bacterial families suggest a functional reorganization of the ecosystem. In SWITCH+EtOH mice, ethanol intake increased the relative abundance of Staphylococcaceae and reduced that of Peptococcaceae compared to the SWITCH group. Staphylococcaceae comprises species tolerant to ethanol and oxidative stress [88, 89], while the decrease in Peptococcaceae points to the loss of cholic acid‐converting bacteria capable of producing hydrophobic secondary bile acids via 7α‐dehydroxylation [90, 91]. This change aligns with the increase in cholate detected in the metabolomic profile, indicating partial suppression of this microbial pathway. In contrast, the abundance of Bifidobacteriaceae, which was elevated in SWITCH compared to CTRL, remained unaffected by ethanol. The persistence of these saccharolytic and ethanol‐tolerant strains, which generate acetate and lactate, may partially compensate for epithelial barrier disruption [92]. Although suggestive, the cecal microbiota‐linked associations described above remain fundamentally correlational. Future studies should establish causality using microbiota transfer, germ‐free models, selective depletion of specific microbial taxa, or supplementation with defined metabolites to delineate alternative mechanisms and consolidate causal inference.

Ethanol exposure significantly reduced the cecal metabolome's free amino acids and γ‐glutamyl derivatives. This profile suggests reduced protein‐fermenting and peptidase‐producing bacteria, impairing dietary protein degradation and amino acid release into the lumen [93, 94, 95]. Alternatively, luminal depletion may stem from enhanced proximal absorption of amino acids recruited for glutathione synthesis in response to ethanol‐induced oxidative stress [96, 97, 98]. These findings align with Xie et al., who reported sharp amino‐acid reductions in the cecum and colon of ethanol‐treated rats [99]. The authors attributed these alterations to ethanol‐induced intestinal dysbiosis and diminished intestinal and hepatic catabolism [99]. Piacentino et al. reported similar reductions in non‐human primates, associated with dysbiosis, oxidative stress, and increased catabolism [100]. Dedon et al. observed consistent results in humans, who reported decreased fecal amino acid levels associated with an enrichment of microbial catabolic pathways and markers of intestinal inflammation and oxidative stress [39]. Lower γ‐glutamyl derivatives may reflect either diminished γ‐glutamyl‐transferase activity or accelerated host utilization of these peptides to replenish cysteine and glutathione during redox challenge [101, 102, 103, 104, 105]. In SWITCH+EtOH mice, increased cytosine and reduced cytidine 5′‐monophosphate levels suggest upregulation of the pyrimidine salvage pathway, a metabolic adaptation to oxidative stress [106]. The concurrent accumulation of cholate, a bile acid commonly modified by gut microbiota, reinforces the presence of dysbiosis. Although synthesized in the liver, excess cholate, as reported in alcoholic liver injury, reflects disruption of the enterohepatic circuit and may aggravate intestinal inflammation [107, 108]. These findings support that, even without overt changes in alpha diversity, ethanol alters microbiota functionality, fostering a pro‐inflammatory state that may worsen hepato‐intestinal dysfunction via the gut‐liver axis, especially in organisms with prior obesogenic exposure.

Alterations in the cecal compartment appear to impact the colonic mucosa. In the SWITCH+EtOH group, Cldn7 expression increased relative to the SWITCH group and returned to control levels in the proximal colon, while ZO‐1 remained reduced across all segments. This imbalance may compromise tight junction stability [76, 109]. The concurrent depletion of polyamines, SCFAs, glycine, and serine observed in SWITCH animals suggests a microenvironment less supportive of epithelial barrier renewal, particularly in the distal colon, where structural protein levels remain low [110]. Additionally, *Lyz* transcription was reduced in SWITCH+EtOH mice, which may reflect impaired mucosal antimicrobial defense [111, 112]. Crypt depth was also reduced in the proximal colon, indicating morphological alterations associated with ethanol exposure [113]. These findings suggest an incompletely restored epithelium, with impaired barrier function and increased vulnerability, especially in distal regions.

In conclusion, our data demonstrate that weight loss after an obesogenic diet does not amount to complete restoration of intestinal or systemic homeostasis. Metabolic memory persists after weight loss and sustains neurobehavioral changes that likely shift the compulsive drive from highly palatable foods to ethanol intake [15, 114]. Concurrently, the cecal microbiome is functionally remodeled. These luminal disturbances reverberate on the colonic mucosa. Such an arrangement likely sustains low‐grade inflammation, weakens the intestinal barrier, and amplifies metabolic and hepatic vulnerability in individuals with an obesogenic history who begin to consume alcohol.

## Author Contributions

Renato Elias Moreira Júnior conducted the animal model and behavioral tests, performed statistical analyses, interpreted the results, processed and evaluated the histological slides, and wrote the initial draft of the manuscript. Mírian Velten Mendes contributed to the interpretation and discussion of the results. Mariana Siqueira Amormino, Gwenaëlle Randuineau, Célia Le Boulenger, Sylvie Guéri, and Véronique Romé contributed to the execution of the experiments. Gaelle Boudry contributed to the execution and coordination of the experiments, performed statistical analyses, interpreted the results, and participated in the discussion and revision of the initial manuscript. Ana Lúcia Brunialti‐Godard coordinated and conducted the experiments, interpreted the results, participated in their discussion, supervised the implementation of the animal model, and supervised the manuscript development phase. All authors contributed to the review and editing of the manuscript and approved the final version.

## Conflicts of Interest

The authors declare no conflicts of interest.

## Supporting information


**Table S1:** Experimental diet compositions (g/kg diet).

**Table S2:** primers (5′ → 3′) used for RT‐qPCR.

**Figure S1:** Ethanol consumption and preference in CTRL+EtOH and SWITCH+EtOH groups. (A) Ethanol intake (g/kg/24 h). (B) Ethanol preference (%). Statistical analysis: (A) Two‐way repeated measures ANOVA followed by Sidak's post hoc test; (B) One‐sample *t*‐test comparing ethanol preference to the hypothetical value of 50.1%. Bars represent mean ± SEM. *p < 0.05, **p < 0.01, ***p < 0.001, ****p < 0.0001.

## Data Availability

The data supporting the findings of this study are available within the article in the Materials and Methods, Results, and/or Supporting Information sections. Raw data are available from the corresponding author upon reasonable request.
