# Supplementary material for: Transition From a High‐Sugar and Butter to a Standard Diet Leads to Cecal Dysbiosis, Disrupts Intestinal Homeostasis, and Favors Increased Ethanol Consumption and Preference
Source: FASEB J. 2025 Oct 8;39(19):e71105. doi: 10.1096/fj.202502123R (PMC12506847; doi:10.1096/fj.202502123R)
Supplement: Supplementary file 1 — Table S1: Experimental diet compositions (g/kg diet). [file FSB2-39-e71105-s001.docx]

**Supplementary Table 1**: Experimental diet compositions (g/kg diet)

| **Ingredient** | AIN93G [1] | HSB [2] | Role/Function |
| --- | --- | --- | --- |
| Casein | 200.0 | 200.0 | Protein source |
| Corn starch | 397.5 | 208.6 | Carbohydrate |
| Sugar | 100.0 | 232.0 | Carbohydrate |
| Destrinized starch | 132.0 | 0.0 | Carbohydrate |
| Commercial butter | 0.0 | 189.0 | Saturated-fat source |
| Soybean oil | 70.0 | 70.0 | Essential fatty acids |
| Cellulose | 50.0 | 50.0 | Fiber |
| Mineral mix (AIN-93G) | 35.0 | 35.0 | Minerals |
| Vitamin mix (AIN-93G) | 10.0 | 10.0 | Vitamins |
| L-Cystine | 3.0 | 3.0 | Sulfur amino acid |
| Choline bitartrate | 2.5 | 2.5 | Methyl donor |
| BHT | 0.014 | 0.014 | Antioxidant |
| **Total mass (g)** | 1000 | 1000 |  |
| **Energy density (Kcal/g)** | 3.9 | 4.9 |  |

**HSB** - High Sugar and Butter; **AIN93G** - American Institute of Nutrition 1993 Growth

**REFERENCES**

1. Reeves, P.G., F.H. Nielsen, and G.C. Fahey, *AIN-93 purified diets for laboratory rodents: final report of the American Institute of Nutrition ad hoc writing committee on the reformulation of the AIN-76A rodent diet.* J Nutr, 1993. **123**(11): p. 1939-51. DOI: [10.1093/jn/123.11.1939](https://doi.org/10.1093/jn/123.11.1939)

2. Maioli, T.U., et al., *High sugar and butter (HSB) diet induces obesity and metabolic syndrome with decrease in regulatory T cells in adipose tissue of mice.* Inflamm Res, 2016. **65**(2): p. 169-78. DOI: [10.1007/s00011-015-0902-1](https://doi.org/10.1007/s00011-015-0902-1)
