# Supplementary material for: Transition From a High‐Sugar and Butter to a Standard Diet Leads to Cecal Dysbiosis, Disrupts Intestinal Homeostasis, and Favors Increased Ethanol Consumption and Preference
Source: FASEB J. 2025 Oct 8;39(19):e71105. doi: 10.1096/fj.202502123R (PMC12506847; doi:10.1096/fj.202502123R)
Supplement: Supplementary file 2 — Table S2: primers (5′ → 3′) used for RT‐qPCR. [file FSB2-39-e71105-s002.docx]

**Supplementary Table 2:** primers (5'→3') used for RT-qPCR

| ***Gene*** | **Forward** | **Reverse** | **Reference** |
| --- | --- | --- | --- |
| Colon | | | |
| *Sod2* | TAACGCGCAGATCATGCAGCTG | AGGCTGAAGAGCGACCTGAGTT | [1] |
| *Cat* | AGCGACCAGATGAAGCAGTG | TCCGCTCTCTGTCAAAGTGTG | [1] |
| *Sod1* | GGAAGCATGGCGATGAAAGC | GCCTTCTGCTCGAAGTGGAT | [2] |
| *Nqo1* | CTCTCAAACCAGCCTTTCAG | CAACCCCATCATTTCCAG | [1] |
| *Gsr* | GACACCTCTTCCTTCGACTACC | CACATCCAACATTCACGCAAG | [1] |
| *Gpx2* | GAGAACGGGTCATCATAAGG | TCGGACATCAGGAGAACTG | [1] |
| *Duox2* | GCCCTCAAAGTAGTCAATCAC | GGACCTCTATTCAGCAACATC | [1] |
| *Tlr9* | TCCATCACCTGAGCCATCTG | TAGGTCCAGCACCGAGAGGT | [3] |
| *Tlr4* | AGTAGCACTGACACCTTCCTT | GCCTTAGCCTCTTCTCCTTCA | [4] |
| *Tlr2* | GTCACCATGGCCAATGTAGG | GCCTCCCTCTCATCAGTTCT | [5] |
| *Lyz* | GGAATGGATGGCTACCGTGG | CATGCCACCCATGCTCGAAT | [6] |
| *Defa6* | GGACCAGGCTGTGTCTGTCT | TTGCAGCCTCTTGCTCTACA | [6] |
| *Reg3g* | TTCCTGTCCTCCATGATCAAA | CATCCACCTCTGTTGGGTTC | [7] |
| *Alpi* | CCAGCAGTAACTCACCTCATGG | GAAGCCTTGTGGATTCCTGCTG | [8] |
| *Muc2* | TGTGGAACCGGGAAGATG | GACCACAGGTATGGTTCTGGA | [9] |
| *ChgA* | CGATCCAGAAAGATGATGGTC | CGGAAGCCTCTGTCTTTCC | [9] |
| *Ephb2* | GCGGCTACGACGAGAACAT | GGCTAAGTCAAAATCAGCCTCA | [1] |
| *Ki67* | CTGCCTGCGAAGAGAGCATC | AGCTCCACTTCGCCTTTTGG | [10] |
| *LgR5* | CCTGTCCAGGCTTTCAGAAG | CAGAGGCGATGTAGGAGACTG | [9] |
| *Tjp1* | GATCATTCCACGCAGTCTCC | GGCCCCAGGTTTAGACATTC | [5] |
| *Ocln* | ATGTCCGGCCGATGCTCTC | TTTGGCTGCTCTTGGGTCTGTAT | [1] |
| *Cldn7* | GGCCACTCGAGCCTTAATGGTG | CCTGCCCAGCCGATAAAGATGG | [1] |
| *Cldn3* | CGTACAAGACGAGACGGCCAAG | CACGTACAACCCAGCTCCCATC | [1] |
| *Cldn2* | AAGGTGCTGCTGAGGGTAGA | AGTGGCAGAGATGGGATTTG | [1] |
| *Hprt1* | TCAGTCAACGGGGGACATAAA | GGGGCTGTACTGCTTAACCAG | [2] |
| *Gapdh* | TGTGTCCGTCGTGGATCTGA | TTGCTGTTGAAGTCGCAGGAG | [11] |
| Striatum | | | |
| *Drd1* | GAGTCGGGGAGTGGTCT3 | CAATCTCAGTCACTTTTCGGG | [12] |
| *Drd2* | GCCAACCTGAAGACACCACT | CTTGACAGCATCTCCATTTCC | [12] |
| *Slc6a3* | TTCCGAGAGAAACTGGCCTA | TGTGAAGAGCAGGTGTCCAG | [13] |
| *Comt* | GTGCTTTGAAGATGCCGGAG | GTGTGCTTTGCATTTAGGACA | This study |
| *Actb* | GTGGGAATGGGTCAGAAGG | CCAGTGCAAGTGCTGAAGAG | [14] |
| *Gapdh* | AGGAGCGAGACCCCACTAAC | GGTCATCTTTTCACGGTTGG | [14] |

*Sod2* (superoxide dismutase 2), *Cat* (catalase), *Sod1* (superoxide dismutase 1), *Nqo1* (NAD(P)H dehydrogenase [quinone] 1), *Gsr* (glutathione‑disulfide reductase), *Gpx2* (glutathione peroxidase 2), *Duox2* (dual oxidase 2), *Tlr9* (Toll-like receptor 9), *Tlr4* (Toll-like receptor 4), *Tlr2* (Toll-like receptor 2), *Lyz* (lysozyme C), *Defa6* (defensin alpha 6), *Reg3g* (regenerating islet-derived protein 3 gamma), *Alpi* (intestinal alkaline phosphatase), *Muc2* (mucin 2), *Chga* (chromogranin A), *Ephb2* (Eph receptor B2), *ki67* (Ki-67 antigen), *Lgr5* (leucine-rich repeat-containing G protein-coupled receptor 5), *Tjp1* (tight junction protein 1), *Ocln* (occludin), *Cldn7* (claudin 7), *Cldn3* (claudin 3), *Cldn2* (claudin 2), *Hprt1* (hypoxanthine phosphoribosyltransferase 1), *Gapdh* (glyceraldehyde-3-phosphate dehydrogenase), *Drd1* (dopamine receptor D1), *Drd2* (dopamine receptor D2), *Slc6a3* (solute carrier family 6 member 3), *Comt* (catechol-O-methyltransferase) and *Actb* (actin beta).

**REFERENCES**

1. Guerbette, T., et al., *Bioenergetic adaptations of small intestinal epithelial cells reduce cell differentiation enhancing intestinal permeability in obese mice.* Mol Metab, 2025. **92**: p. 102098.

2. Novak, S., et al., *Anti-Inflammatory Effects of Hyperbaric Oxygenation during DSS-Induced Colitis in BALB/c Mice Include Changes in Gene Expression of.* Mediators Inflamm, 2016. **2016**: p. 7141430.

3. Wong, D.V.T., et al., *TLR4 deficiency upregulates TLR9 expression and enhances irinotecan-related intestinal mucositis and late-onset diarrhoea.* Br J Pharmacol, 2021. **178**(20): p. 4193-4209.

4. Islam, T., et al., *Curcumin Reduces Adipose Tissue Inflammation and Alters Gut Microbiota in Diet-Induced Obese Male Mice.* Mol Nutr Food Res, 2021. **65**(22): p. e2100274.

5. Zhang, X.S., et al., *Maternal cecal microbiota transfer rescues early-life antibiotic-induced enhancement of type 1 diabetes in mice.* Cell Host Microbe, 2021. **29**(8): p. 1249-1265.e9.

6. Yang, W., et al., *Translocation and Dissemination of Gut Bacteria after Severe Traumatic Brain Injury.* Microorganisms, 2022. **10**(10).

7. Shi, H., et al., *β-glucan attenuates cognitive impairment via the gut-brain axis in diet-induced obese mice.* Microbiome, 2020. **8**(1): p. 143.

8. Jin, Z., et al., *Roux-en-Y gastric bypass potentially improved intestinal permeability by regulating gut innate immunity in diet-induced obese mice.* Sci Rep, 2021. **11**(1): p. 14894.

9. Anandam, K.Y., et al., *Molecular mechanisms involved in the adaptive regulation of the colonic thiamin pyrophosphate uptake process.* Am J Physiol Cell Physiol, 2017. **313**(6): p. C655-C663.

10. Wang, T., et al., *Scutellarin Alleviates Bone Marrow Mesenchymal Stromal Cellular Senescence via the Ezh2-Nrf2 Signalling Axis in Diabetes-Induced Bone Loss.* Cell Prolif, 2025. **58**(4): p. e13790.

11. Zhang, J., et al., *Dynamic changes of CX3CL1/CX3CR1 axis during microglial activation and motor neuron loss in the spinal cord of ALS mouse model.* Transl Neurodegener, 2018. **7**: p. 35.

12. Martins de Carvalho, L., et al., *High-fat diet withdrawal modifies alcohol preference and transcription of dopaminergic and GABAergic receptors.* J Neurogenet, 2018: p. 1-11.

13. Júnior, R.E.M., et al., *Behavioral changes and transcriptional regulation of mesolimbic dopaminergic genes in a mouse model of binge eating disorder by diet intermittent access.* J Nutr Biochem, 2025. **135**: p. 109784.

14. Bibancos, T., et al., *Social isolation and expression of serotonergic neurotransmission-related genes in several brain areas of male mice.* Genes Brain Behav, 2007. **6**(6): p. 529-39.
