# Supplementary material for: Transition From a High‐Sugar and Butter to a Standard Diet Leads to Cecal Dysbiosis, Disrupts Intestinal Homeostasis, and Favors Increased Ethanol Consumption and Preference
Source: FASEB J. 2025 Oct 8;39(19):e71105. doi: 10.1096/fj.202502123R (PMC12506847; doi:10.1096/fj.202502123R)
Supplement: Supplementary file 3 — Figure S1: Ethanol consumption and preference in CTRL+EtOH and SWITCH+EtOH groups. (A) Ethanol intake (g/kg/24 h). (B) Ethanol preference (%). Statistical analysis: (A) Two‐way repeated measures ANOVA followed by Sidak's post hoc test; (B) One‐sample t‐test comparing ethanol preference to the hypothetical value of 50.1%. Bars represent mean ± SEM. *p < 0.05, **p < 0.01, ***p < 0.001, ****p < 0.0001. [file FSB2-39-e71105-s003.docx]

**CTRL Animals Exhibit Low Ethanol Intake and Preference in the Two-Bottle Choice Paradigm**

Control animals subjected to the two-bottle free-choice paradigm between water and ethanol (CTRL+EtOH; n = 6) did not exhibit high intake or ethanol preference. Ethanol consumption and preference were assessed in CTRL+EtOH animals in comparison to the SWITCH+EtOH group. Throughout T2, CTRL+EtOH animals consumed significantly less ethanol than the SWITCH+EtOH group (p < 0.005). Moreover, they showed a lower ethanol preference (p < 0.0001). When compared to the hypothetical high-preference threshold of 50.1%, CTRL+EtOH animals exhibited an aversion to ethanol (t = 13.99, df = 5, p < 0.001), whereas the opposite pattern was observed in the SWITCH+EtOH group.


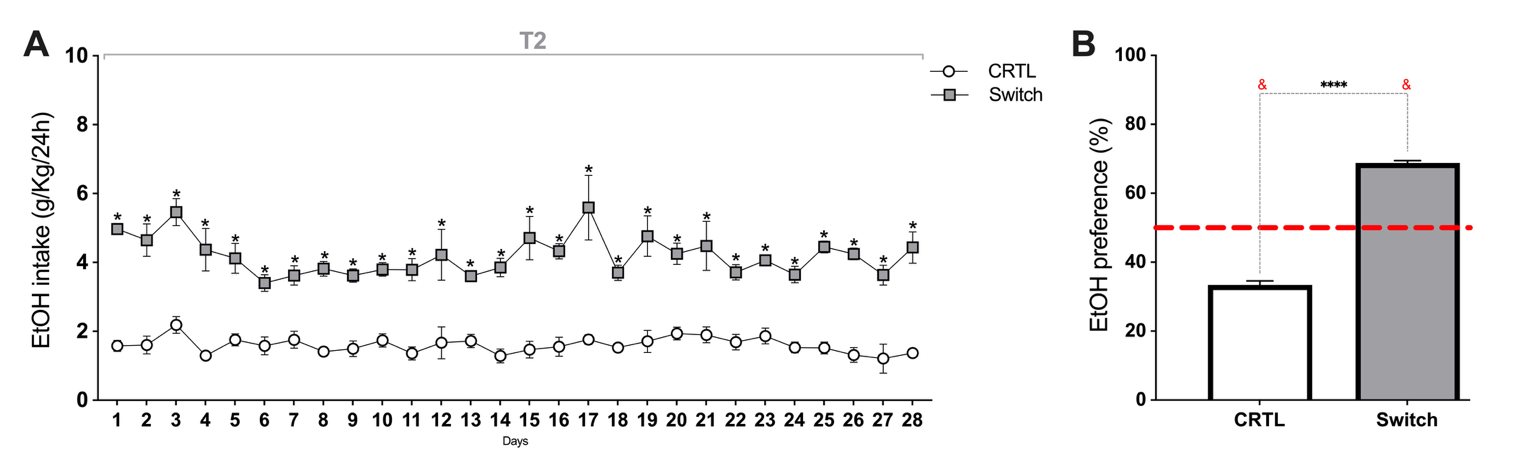


**Supplementary Figure 1**. Ethanol consumption and preference in CTRL+EtOH and SWITCH+EtOH groups. (A) Ethanol intake (g/kg/24 h). (B) Ethanol preference (%). Statistical analysis: (A) Two-way repeated measures ANOVA followed by Sidak’s post hoc test; (B) One-sample t-test comparing ethanol preference to the hypothetical value of 50.1%. Bars represent mean ± SEM. *P < 0.05, **P < 0.01, ***P < 0.001, ****P < 0.0001.
